# Interferon-λs and Plasmacytoid Dendritic Cells: A Close Relationship

**DOI:** 10.3389/fimmu.2017.01015

**Published:** 2017-08-23

**Authors:** Giulia Finotti, Nicola Tamassia, Marco A. Cassatella

**Affiliations:** ^1^Department of Medicine, Section of General Pathology, University of Verona, Verona, Italy

**Keywords:** plasmacytoid dendritic cells, interferon lambda, innate immunity, IFNα, IL-3, CXCL10, TNFα

## Abstract

Interferon lambdas (IFNλs) are recently discovered cytokines acting not only at the first line of defense against viral infections but also at the mucosal barriers. In fact, a peculiar feature of the IFNλ system is the restricted expression of the functional IFNλR, which is known to be limited to epithelial cells and discrete leukocyte subsets, including the plasmacytoid dendritic cells (pDCs). In the latter case, current data, discussed in this minireview, indicate that IFNλs positively regulate various pDC functions, including pDC expression of interferon-dependent gene (ISG) mRNAs, production of cytokines, survival, and phenotype. Although the knowledge of the effects on pDCs by IFNλs is still incomplete, we speculate that the peculiar pDC responsiveness to IFNλs provide unique advantages for these innate immune cells, not only for viral infections but also during autoimmune disorders and/or tumors, in which pDC involvement and activation variably contribute to their pathogenesis.

## Introduction

Human dendritic cells (DCs) in the blood typically include the myeloid DCs (mDCs), enlisting the BDCA1^+^/CD1c^+^ and BDCA3^+^/CD141^+^ DCs, as well as the plasmacytoid DCs (pDCs) (1). All peripheral DCs originate from a common DC progenitor (2) and act as antigen-presenting cells (APCs) to initiate adaptive immune responses (3). Among DCs, pDCs are distinguishable given their peculiar phenotype, tissue localization, and specialized functions (4). pDCs constitute 0.2–0.6% of the peripheral blood mononuclear cells (PBMCs) in healthy individuals (5) and are specialized in the production of type I interferon (IFN) (6–8). Human pDCs specifically express the C-type lectin BDCA2/CD303 molecule, the alpha chain of the interleukin-3 receptor (IL-3Rα/CD123), and neuropilin-1/BDCA4 (9), but not CD11c, which is instead expressed by mDCs (1, 3). Under steady state conditions, pDCs localize in the T cell areas of the lymph nodes (LNs), while they are undetectable in almost all peripheral tissues (5, 10). Migration of pDCs into LNs and inflamed tissues involves discrete adhesion molecules (CD62L, PSGL-1, β1- and β2-integrin), as well as activated chemokine receptors, including CXCR3, CXCR4, CCR2, CCR5, and CCR7 (11, 12). Once recruited into tissues, pDCs orchestrate immune responses, as well as interact with, activate, or are activated by T, B, NK cells, and other leukocytes (4, 13, 14).

Plasmacytoid dendritic cells are specialized in recognizing viral and/or self/non-self nucleic acids, for instance through TLR7 and TLR9, to ultimately produce IFNα following an intracellular signaling cascade activating interferon regulatory factor 7 (IRF7) (15). IFNα, in turn, not only induces the transcription of interferon-dependent genes (ISGs) to limit the spread of viral pathogens (16) but also amplifies immune responses by modulating selected functions of NK, myeloid, B and T cells (17, 18). TLR7/9 engagement also leads pDCs to differentiate into mature cells, thus acquiring a more DC morphology and APC capacity (5, 19, 20). Similar effects on pDCs are observed in response to IL-3, a cytokine also known to maintain pDCs alive (10). Accordingly, TLR and/or IL-3-stimulated pDCs upregulate the expression of MHC-II and costimulatory molecules (including CD80, CD86, and CD40), as well as produce both proinflammatory cytokines (TNFα and IL-6) and chemokines (CCL4, CCL5, CXCL9, and CXCL10) (7, 11, 13, 21). Notably, endogenous TNFα concurs to pDC maturation (22), while autocrine/paracrine IFNα promotes the survival of pDCs *via* induction of antiapoptotic genes (23). Activated/mature pDCs, in turn, become able to promote the polarization of T helper lymphocytes into Th1, Th2, Th17, or also Treg cells, depending on the context (7, 8, 10, 24).

Plasmacytoid dendritic cells also produce type III IFNs/IFNλs (25), for instance in response to HSV (26–28), Sendai virus (27), Flu (27), Imiquimod/R837 (synthetic TLR7 ligands) (26–29), CpG oligodeoxyribonucleotides (26–28, 30–32), or upon cocolture with hepatitis C virus (HCV)-infected Huh7.5 (30, 31). The IFNλ family includes four members, three of them identified in 2003 (e.g., IFNλ1/IL-29, IFNλ2/IL-28A, and IFNλ3/IL-28B), the fourth one (IFNλ4), which shares only ~30% identity with other IFNλs, but signals through the same receptor complex, discovered more recently (2013) (33). IFNλs not only display potent antiviral activities (34–36) but also exert other effects involved in autoimmunity and tumor progression (37, 38). Moreover, it has become increasingly clear that IFNλs evolved to serve as a first line of defense at the mucosal barrier, particularly at the level of the respiratory and gastrointestinal tracts, which are the initial target of most invasive pathogens (36). In fact, a peculiarity of the IFNλ system is the restricted distribution of the IFNλR (39–41), which consists of a specific IFNλR1 chain (also known as IL-28R), and the ubiquitously expressed IL10R2 chain (40, 41). Epithelial cells of the intestine, lungs, skin, and liver constitutively express the IFNλR1 chain and thus represent the primary targets of IFNλs (42). In such regard, there has been a great interest in specifically characterizing the antiviral role of IFNλs during HCV and hepatitis B virus infections (43–47). In the former case, in fact, although not yet explained in the context of HCV pathogenesis, several genome-wide association studies have demonstrated a link between single-nucleotide polymorphisms near the IFNλ3 and IFNλ4 genomic loci and either the spontaneous clearance or the sustained response to IFNλ-treatment in HCV-infected patients (48–50). Moreover, IFNλ1 has been used for clinical trials in HCV patients (51) confirming an antiviral efficacy equivalent to IFNλ, but with less toxicity (51). Fibroblasts, splenocytes, bone marrow (BM)-derived macrophages, and endothelial cells do not express IFNλR1 and thus do not respond to IFNλs (42, 52, 53). Among human leukocytes, only pDCs and, less prominently, B cells, have been shown to constitutively express a complete IFNλR (26, 27). Consistently, IFNλs have been shown to trigger phosphorylation of STAT1 (27, 54, 55), STAT2 (54), STAT3, and STAT5 (55), in either freshly isolated pDCs (54) or pDCs gated among total PBMCs (27, 55), as well as various functional responses herein summarized.

## Production of Cytokines by pDCs Incubated with IFNλs

Interferon lambdas have been described to stimulate the production of cytokines and chemokines in pDCs. We reported that human pDCs incubated for up to 42 h with 30 IU/ml IFNλ1 or IFNλ3 produce variable, but significant, levels of CXCL10, usually (but not always) followed by IFNα (54). Consistently, experiments using anti-IFNαR antibodies only partially blocked CXCL10 derived from pDCs incubated with IFNλ3 for 42 h (54). Notably, healthy donors could be categorized into two groups based on the levels of IFNα produced by their IFNλ3-treated pDCs [e.g., very modest ≤150 pg/ml/42 h: elevated ≥500 pg/ml/42 h] (54). By similar criteria, referred instead to CXCL10, healthy donors could be independently divided into three groups: one having pDCs producing modest quantities of CXCL10 (ranging from 22 ± 11 pg/ml/18 h to 163 ± 24 pg/ml/42 h); another one, having pDCs producing elevated CXCL10 levels already after 18 h (865 ± 297 pg/ml) without further increasing thereafter; and a third one, having pDCs producing maximal CXCL10 levels after 42 h of IFNλ3-treatment (1,320 ± 264 pg/ml) (54). It should be pointed out that such an extremely variable production of both IFNα and CXCL10 were shown not to depend on differences in the viability of pDCs among the donor groups. Moreover, the patterns of CXCL10 production by pDCs somewhat recalled previous data (56), likely attributable to pDCs, in which PBMCs from healthy donors were described to function either as “early” or as “late” responders to 3,500 IU/ml IFNλ1, depending, respectively, on the more rapid or more delayed kinetics of CXCL9, CXCL10, and CXCL11 transcript induction. Whatever the case is, the molecular bases underlying the variable capacity of pDCs to produce IFNα and CXCL10 by the different donor typologies, as well as their potential biologic implications, require further investigations.

In addition to CXCL10 and IFNα, we also detected low but biologically active amounts of TNFα in supernatants harvested from purified pDCs incubated with IFNλ3 (54). In fact, experiments in which supernatants from IFNλ3-treated pDCs were transferred to CD14^+^-monocytes in the presence or absence of reagents inhibiting TNFα, namely etanercept (ETA) and adalimumab, revealed that they induced CCL4 and IκBα mRNA expression in a TNFα-dependent manner (54). It should be pointed out that, in contrast with our results, 3,500 IU/ml IFNλ1-treated PBMCs were previously found able to produce CXCL8, IL-6, and IL-10, but not TNFα or IL-1α (57), possibly because of the short stimulation period. Similarly, Flt3-generated BM-derived murine pDCs incubated with IFNλ2 were found unable to produce CXCL10 and IL-6 (58). However, whether Flt3-generated BM-derived murine pDCs express the complete IFNλR, or whether their blood counterpart behaved as human pDCs, was not reported.

Because flow cytometry experiments uncovered that both IFNλ3 and IL-3 increase the levels of surface CD123 and IFNλR1 in human pDCs (54, 59), in a subsequent study, we investigated whether IFNλ3 and IL-3 together could promote stronger pDC responses. This was found to be the case, as we could show that 30 IU/ml IFNλ3 and 20 ng/ml IL-3 induce in pDCs a synergistic production of both IFNα and TNFα (59). Moreover, endogenously produced TNFα was found to almost completely control the synergistic production of IFNα in IFNλ3 plus IL-3-treated pDCs (59). Under the same experimental conditions, or in pDCs incubated with IFNλ3 only, endogenously produced IFNα did not drive ISG mRNA expression, unlike its effect in IL-3-treated pDCs. On the other hand, endogenous TNFα was found to drive ISG mRNA expression in both IFNλ3- and IL-3-stimulated pDCs (59).

## Expression of ISG mRNAs and Phosphorylation of STATs in IFNλ-Treated pDCs

Plasmacytoid dendritic cells have been shown to *de novo* express a variety of ISG mRNAs in response to IFNλs, which further support the protective role of the IFNλ/pDC system in viral infections. For example, 2’-5’-oligoadenylate synthetase 1 (OAS1) and IRF7 mRNAs were found as induced in murine pDCs incubated with 100 ng/ml IFNλ2 (52). In humans, we and others have reported that both IFNλ1 and IFNλ3 induce the mRNA expression of MX dynamin like GTPase 1 (MX1) (59, 60), protein kinase R (PKR), interferon induced protein with tetratricopeptide repeats 1 (IFIT1), ISG ubiquitin-like modifier (ISG15), and C-X-C motif chemokine ligand 10 (CXCL10) (54, 55, 59). Our unpublished observations prove that also CXCL9, TLR7, IFIT2, and TNF-related apoptosis inducing ligand (TRAIL) are induced by IFNλ3 in human pDCs. All these mRNAs were shown to reach maximal levels after 18 h of incubation of pDCs treated with 30 IU/ml IFNλ1 or IFNλ3 (54). Experiments conducted in pDCs preincubated in the presence of anti-IFNαR antibodies, and then cultured with IFNλ3 plus IL-3, which, at the 18 h-time point, express and release much higher levels of, respectively, ISG mRNAs and IFNα, than pDCs incubated with IFNλ3 alone (59), revealed that endogenous IFNα is minimally involved in autocrinally activating ISG mRNA expression (59). Consistently, and even though IFNα is typically considered more potent than IFNλ in inducing ISG gene expression, we observed that equivalent concentrations of IFNλ3 and IFNα (e.g., 30 IU/ml) induce, in human pDCs, comparable levels of STAT1 and STAT2 phosphorylation and ISG15, IFIT1, and MX1 transcripts (our unpublished observations). However, we also noticed that kinetics of both STAT phosphorylation and ISG mRNA induction were more accelerated in response to IFNα than IFNλ3, consistent with studies in other cells (61–63). It should be also pointed out that, in a previous study, the levels of MX1 mRNA induced by IFNα in purified pDCs were found to be higher than those induced by IFNλ3 (60), but IFNα was used at concentrations approximately 10-fold higher than IFNλ3 (1,000 vs 100 IU/ml, respectively). Under similar experimental conditions, only IFNα, but not IFNλs, was shown to activate STAT6 phosphorylation in purified pDCs (55), independently from the concentrations used.

Recent evidence suggests that, under specific experimental settings, IFNα/β and IFNλ control gene expression, as well as contribute to the antiviral state, by using different and non-redundant mechanisms. For instance, unlike IFNβ (64), IFNλ1 and IFNλ2 were shown to activate an alternative signaling pathway involving Jak2 in UMUC-3 and Huh7.5 cell lines (64, 65). Similarly, the antiviral activity induced in T84 cell lines by IFNλs, but not IFNα, was found to be strongly dependent on the mitogen-activated protein kinases (MAPKs) activation (66). However, whether IFNλ activates Jak2 and/or MAPK in pDCs is currently unknown.

## IFNλs Promote the Survival of pDCs

Plasmacytoid dendritic cells are known to spontaneously undergo apoptosis when cultured *in vitro* (10, 22). In this context, one of the remarkable effects that IFNλs exert in pDCs freshly purified from the blood is to prolong their survival for up to 42 h (54), similarly to IL-3 (54). While equivalent concentrations of IFNλ1 or IFNλ3 (30 and 100 IU) were found to exert comparable prosurvival activities in pDCs, no further enhancement was observed when IFNλ3 was used in combination with IL-3, indicating that each cytokine produces already the maximal prosurvival effect by itself (59). In additional experiments, we found that both endogenous TNFα and IFNα partially sustain the survival of pDCs cultured in the presence of IFNλ3. Similarly, anti-IFNαR antibodies were found to decrease survival of pDCs incubated with IL-3 alone (our unpublished observations) or CpG-C plus glucocorticoids (23), while TNFα blockers had no or only a slight effect under the same conditions (22, 23). However, no modulation of survival was found by inhibiting both TNFα and IFNα in pDCs cultured with IFNλ3 plus IL-3. Conceptually, our data not only confirm, but further support, previous observations showing that 35–350 IU/ml IFNλ1 counteracts the proapoptotic effects that dexamethasone (DEX) exerts in pDCs present within PBMCs (27). The molecular mechanisms whereby IFNλs promote pDC viability are unknown and should be characterized.

## IFNλs Modulate the Expression of Various Surface Markers in pDCs

In addition to inducing cytokine production and ISG mRNA expression, or promoting survival, IFNλs have been shown to trigger the maturation of pDCs, according to phenotypic changes. For instance, incubation of PBMCs with 35–350 IU/ml IFNλ1 for 7 or 20 h has been shown to weakly increase the surface expression of CD80, ICOS-L, CD62L, CD83, CCR7, and MHC-I, but not of CD86, in CD123^+^/CD303^+^-gated-pDCs (26, 27). By using freshly isolated pDCs, we could confirm that 30–100 IU/ml IFNλ3 potently and persistently (e.g., for up to 42 h) modulates the expression of CD86, HLA-DR, CD123, and CD303, in addition to CD62L and CD83. However, in contrast with the data by Megjugorac et al. (26), we found an upregulation of CD86 upon treatment of pDCs with IFNλ3 for 42 h. Although IFNλ3-mediated effects substantially resembled those induced by IL-3 (54, 59), IFNλ3 appeared significantly less potent in upregulating HLA-DR or CD86 expression, or in downmodulating CD303 and CD62L, consistent with a weaker maturational effect on pDCs. Functionally, only one study (26) has specifically analyzed whether 350 IU/ml IFNλ1-treated pDCs could activate CD4^+^ T cells. Accordingly, it has been reported that cocultures of IFNλ1-treated pDCs with allogenic T cells, activated by PMA/ionomycin, produce reduced levels of IL-10, IL-13, and IFNγ than in the absence of IFNλ1 (26). Whether IFNλ-treated pDCs promote Th1, Th2 or Treg polarization has not been specifically investigated yet.

## Conclusion

As synthetically outlined in this minireview, current data suggest that IFNλ is able to regulate pDC functions at various levels (as summarized in Table 1), including the production of IFNα, CXCL10, and TNFα. Because IFNα has been shown to increase the production of IFNλ by CD141^+^ DCs in response to HCV-infected hepatoma cells or poly-I:C (30), data testify for potential cross talk between pDCs and CD141^+^ DCs *via* the two IFN systems. A strict cross talk between pDCs and B cells has been also described, as B cells are known to enhance IFNα, and possibly IFNλs, production by pDCs, *via* cell–cell contact-dependent mechanisms or soluble factors (14). Conversely, TNFα and CXCL10 secreted by IFNλ-activated pDCs might contribute to, respectively, amplify local inflammatory responses and recruit activated T lymphocytes. On the same line, modulation of pDC membrane markers by IFNλ might influence T cell polarization, either promoting or impairing T cell responses, depending on the context. Thus, *in vitro* experiments suggest that IFNλs could orchestrate complex immune cell interactions by amplifying pDC responses, both directly and indirectly. Since *in vitro* pDCs increase the expression of IFNλR1 in response to IL-3 (59), IFNλ3 (59), or R837 (our unpublished observations), it is likely that this phenomenon also happens at the site of infection in response to viral particles or other stimuli. However, whether IFNλR1 modulation positively or negatively affects pDC response to IFNλ, and, in turn, pDC cross talk with other immune cell subpopulations, is not known. Similarly, even though there are three splice variants of the human IFNλR1 gene, encoding either the full length functional IFNλR1, a soluble IFNλR1, or an IFNλR1 variant lacking a membrane-proximal region of the intracellular domain and expected to be signal-incapable (67), no information is present on how they are regulated in pDCs.

**Table 1:** Biological effects of interferon lambdas in human plasmacytoid dendritic cells (pDCs).

IFNλ type	Dose	Investigated response in pDCs	Outcome	Modality of detection	Reference
IFNλ3	30–100 IU/ml	Modulation of IFNλR expression	Increase of mRNA and surface IFNλR1	Real-time qPCR and flow cytometry	[(59) and our unpublished observations]

IFNλ1, IFNλ2, IFNλ3	35–350 IU/ml	Activation of signaling pathways	Induction of STAT-1, -3, -4, and -5 phosphorylation^a^	Flow cytometry	(27, 55)
IFNλ3	30 IU/ml		Induction of STAT-1 and -2 phosphorylation	Immunoblotting	(54)

IFNλ1	35–350 IU/ml	Modulation of maturation markers	Upregulation of CD80, ICOS-L, CD62L, CD83, MHC-I^a^	Flow cytometry	(26, 27)
IFNλ1, IFNλ3	30–100 IU/ml		Upregulation of HLA-DR, CD123, CD83, CD86, CD303, CD62L	Flow cytometry	(54, 59)

IFNλ1	35–350 IU/ml	Survival	Counteraction of the proapoptotic effect exerted by Dexamethasone^a^	Annexin V/propidium iodide staining and intracellular detection of active caspase-3	(27)
IFNλ1, IFNλ3	30–100 IU/ml		Prosurvival effect	Vybrant DyeCycle Violet stain	(54, 59)

IFNλ1	350 IU/ml	Influence on T cell functions	Inhibition of IL-10, IL-13, and IFNγ production by PMA and ionomycin-activated allogenic T cells	ELISA	(26)

IFNλ1, IFNλ3	30–350 IU/ml	ISG mRNA expression	Induction of MX1, protein kinase R, IFIT1, ISG15, and CXCL10 transcripts	Real-time qPCR	(54, 55, 59, 60)
IFNλ3	30 IU/ml		Induction of IFIT2, TLR7, TRAIL, TNFα, IFNα transcripts	Real-time qPCR	[(54, 59) and our unpublished observations]
IFNλ2	100 ng/ml		Induction of oligoadenylate synthetase 1 and interferon regulatory factor 7 transcripts (mouse pDCs)	Real-time qPCR	(52)

IFNλ1	25 ng/ml	Cytokine production	Enhancement of IFNα production in response to hepatitis C virus-infected hepatoma cells or CpG-A	ELISA	(30)
IFNλ1	35 IU/ml		Priming effect and enhancement of IFNα and IFNλ1/3-positive pDCs in response to HSV^a^	Flow cytometry	(27)
IFNλ1, IFNλ3	30–100 IU/ml		Induction of time-dependent production of CXCL10, IFNα and TNFα	ELISA	(54)
IFNλ3	30 IU/ml		Enhancement of IL-3-induced IFNα and TNFα production	ELISA	(59)

*^a^In these papers, pDCs have been identified as BDCA2^+^/CD123^+^ or Lin^−^/CD123^+^ cells, by flow cytometry, within peripheral blood mononuclear cells previously labeled with a combination of specific antibodies*.

As mentioned, given the peculiar expression of IFNλR1 in hepatocytes, clinical trials of IFNλ1 therapy for HCV infection have confirmed that this cytokine has antiviral effects equivalent to IFNα without the same level of associated toxicity (51). Studies of IFNλ treatment of influenza A virus-infected mice have shown similar results (58). In this context, it would be interesting to determine if, and how, circulating and/or tissue resident pDCs are affected by the IFNλ-treatment. Such knowledge might eventually help clarifying the *in vivo* biologic implication(s) of the variable capacity of pDCs to produce IFNα and CXCL10 by the various donor typologies that we described (54). Regardless, treatment with IFNλs might be also useful in patients with autoimmune disorders. A tissue infiltration by pDCs, as well as a type I IFN signature, has been in fact described in SLE, Sjogren’s syndrome, systemic sclerosis, and psoriasis patients (4). In these diseases, pDCs are chronically activated and contribute to their pathogenesis (4). Moreover, high amounts of IFNλ1 or IFNλ2/3 have been detected, respectively, in skin lesions from psoriasis patients (68) and in serum of SLE patients (69, 70), thus pointing for some roles of IFNλs in these diseases (37, 70). In a mouse model of autoimmune arthritis, treatment with IFNλ reduced neutrophil infiltration in the joints and improved disease outcome (71). Similarly, a protective role for IFNλ in allergic asthma has also been proposed (72). Altogether, data suggest that pDCs and IFNλs can have variable contributions to the pathogenesis of autoimmune disorders and could be used as a therapeutic target by either improving or blocking their activity (73).

**Figure 1:**
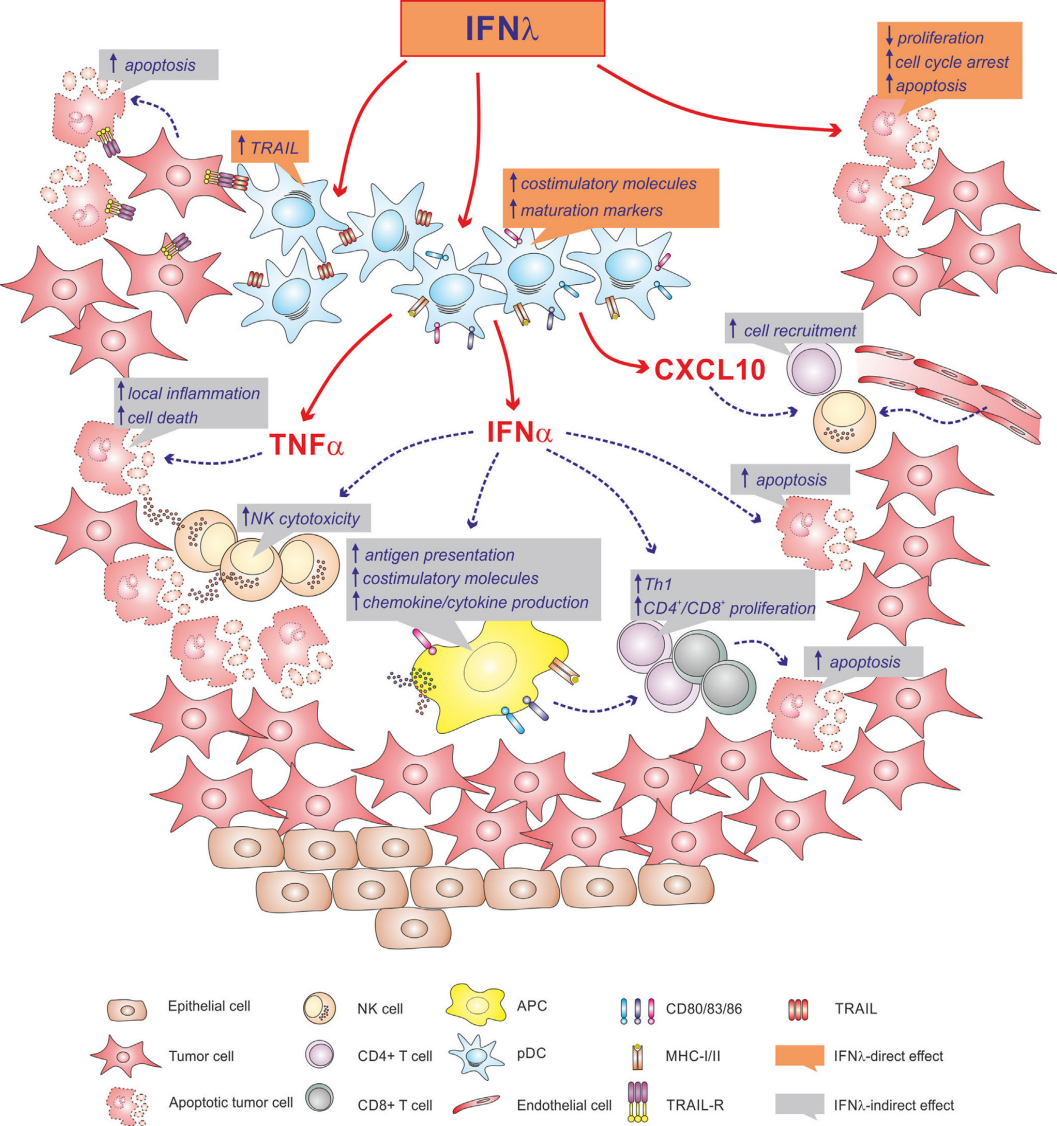
Illustration depicting the potential antitumorigenic role that IFNλs might have within a tumor microenvironment. Accordingly, IFNλs may directly act on tumor cells, may activate local plasmacytoid dendritic cells (pDCs), or may favor the recruitment and activation of immune cells *via* pDC-derived IFNα, TNFα and CXCL10.

Neoplastic cells frequently express IFNλR1 and, after treatment with IFNλs, stop the cell cycle and/or undergo apoptosis (38, 73). In other cases, tumor cells exposed to IFNλs have been shown to become protumorigenic (73). In tumors, infiltration by pDCs is often associated with a poor prognosis, as pDCs tend to be tolerogenic and/or impaired in their functions (4, 74). However, if properly stimulated, pDCs can also promote antitumoral response, for instance, by directly killing tumor cells through TRAIL expression (75), or indirectly *via* IFNα, which mediates NK cell activation. Thus, based on our unpublished observations indicating that IFNλs, in addition to triggering IFNα production, also induce TRAIL mRNA expression in human pDCs, it would be plausible speculating a potential use of IFNλs as adjuvants to chemotherapy regimens (76). Accordingly, IFNλs may induce antitumor activities either by directly acting on tumor cells and intratumor pDCs, or by indirectly favoring the recruitment and activation of immune cells, to ultimately kill tumor cells (Figure 1).

## Author Contributions

GF, NT, and MC have contributed by writing the manuscript.

## Conflict of Interest Statement

The authors declare that the research was conducted in the absence of any commercial or financial relationships that could be construed as a potential conflict of interest.
